# Family-based intervention for patients with type 2 diabetes via WeChat in China: protocol for a randomized controlled trial

**DOI:** 10.1186/s12889-019-6702-8

**Published:** 2019-04-05

**Authors:** Linqi Mao, Jun Lu, Qi Zhang, Yuxi Zhao, Gang Chen, Mei Sun, Fengshui Chang, Xiaohong Li

**Affiliations:** 1 Department of Health Policy and Management, School of Public Health, Fudan University, P.O. Box 177, 130 Dong’an Road, Shanghai, 200032 China; 2 China Research on Disability at Fudan University, Shanghai, 200032 People’s Republic of China; 3 Key Laboratory of Health Technology Assessment, National Health and Family Planning Committee (Fudan University), Fudan University, Shanghai, China; 4 School of Community and Environmental Health, Old Dominion University, Norfolk, Virginia, USA; 5 Department of Health Law and Inspection, School of Public Health, Fudan University, Shanghai, China

**Keywords:** Type 2 diabetes, WeChat, Family member, Randomized controlled trial, China

## Abstract

**Background:**

China has the largest number of cases of diabetes with a high rate of uncontrolled blood sugar. Many studies show that family members’ involvement is related to better glycemic control. However, there is a significant problem with low participation of family members. The widespread use of WeChat provides an opportunity for family members to support their diabetic loved ones in their type 2 diabetes self-management practices. The main aim of this study is to examine the effectiveness of a family-based intervention via WeChat.

**Methods:**

A parallel, two-group, randomized controlled trial will be conducted in the central urban area of Jiading district in Shanghai, China. A total of 222 type 2 diabetics will be randomly divided into an intervention group or a control group using a 1:1 ratio. Patients in the intervention group will receive the usual care, and their family will get education in diabetes control and the importance of family support by subscribing to the WeChat public account. Both the patients and the family members will be followed up at 12 months after the intervention commences. Data collection is scheduled at baseline, 6-months, and 12-months.

**Discussion:**

Family involvement based on WeChat may generate ongoing support for type 2 diabetic patients and improve these patients’ health outcomes. A successful outcome of this study may also provide inspiration for other efforts to provide health education via WeChat.

**Trial registration:**

ChiCTR1900020736. Registered 15 January 2019.

**Electronic supplementary material:**

The online version of this article (10.1186/s12889-019-6702-8) contains supplementary material, which is available to authorized users.

## Background

Diabetes is a major contributor to cardiovascular diseases and a common cause of disability worldwide. Today nearly half a billion people are estimated to be living with the disease, although 50.0% remain undiagnosed [1]. In China, according to national surveys, the prevalence of diabetes has increased rapidly during the past few decades, from 0.9% in the 1980s to 10.9% today [2, 3]. The country has 121 million people with diabetes, which means one in four adults in China is a diabetic. Among these patients, however, only 36.5% have been diagnosed. The direct medical costs of diabetes is expected to be $47.2 billion in 2030 [4].

Glycemic control is one of the main objectives of treatment for diabetics since it is important for preventing and delaying diabetes-related complications. However, the 2013 national survey in China shows that only 39.7% of diagnosed patients were considered to have adequate glycemic control [3]. One possible reason may be poor adherence to diabetes self-management (DSM) behaviors and bad lifestyles among the patients with type 2 diabetes, especially among the elderly [5, 6].

Type 2 diabetes has been one of the leading non-communicable diseases in China. The Chinese Government launched a health management program for type 2 diabetes as a part of their national basic public health services in 2009 wherein diabetic patients could apply for registration in the health management system of their local health community centers. By the end of 2016, the number of registered diabetes patients had reached 27.81 million [7]. The National Guideline for Basic Public Health Services clearly state that the health community centers should provide services to diabetics such as blood glucose testing, physical examination, health education, and nutrition guidance [8]. Health education is considered to be a routine means to promote patients’ DSM. However, due to patients’ poor understanding and co-operation, the service is not effectively implemented, as shown by the failure to raise patients’ awareness of the necessity of glycemic control and to improve their lifestyle [9, 10].

Besides continuous medical care, diabetes requires continuous DSM behaviors and healthy lifestyles [11]. However, the effects of interventions aimed to improve the patients’ health-related behaviors are often inadequate and unsustainable [12]. One possible reason for this may be the failure to use social influences, such as social support, which are crucial for managing chronic conditions. Many studies have explored interventions based on family members’ participation, indicating that family members’ involvement in DSM is helpful to improve the patients’ glycemic control, knowledge related to diabetes, self-efficacy, and quality of life [13–18]. However, based on our literature review, very few studies in China have focused specifically on family members of diabetics [19, 20]. A multinational survey shows that only 25% of family members reported having attended diabetes programs [21]. Thus, the main obstacle to applying a chronic illness support model is the low participation rate of family members [22]. There may be several reasons for this lack of involvement. Family-based intervention usually consists of group sessions. Family members, especially young members, can hardly participate in programs requiring them to visit the appointed meeting place for long periods of time. Just as problematic are home visits, which are not sustainable and are hard to implement in daily practice, because health workers in health community centers already have heavy workloads [23].

The widespread use of smart phones in China, especially among the young, offers an opportunity to remove these obstacles in the implementation of family-based interventions. In China, WeChat is the most popular social networking platform, which is a free application characterized by high convenience and accessibility, presenting complex information by video and graphics, providing a feasible way to spread health information for the public [24]. Many studies show that WeChat has great potential in health intervention [25–29]. However, to our knowledge, few well-designed studies have focused on the combination of a WeChat-based intervention and the involvement of family members for those with type 2 diabetes mellitus in the community. This article is a study protocol for a randomized controlled trial for a family-based intervention via WeChat in two communities within urban China.

### Objectives

Our aim is to implement and evaluate a family-based intervention via WeChat for people with poorly controlled type 2 diabetes within an urban community.

1. To examine the effectiveness of a family-based intervention via WeChat on clinical, psychosocial, and behavioral outcomes for patients with poorly controlled type 2 diabetes.
2. To explore the feasibility and sustainability of this intervention and assess the process of participating in it.

## Methods

### Design and setting

**Fig. 1:**
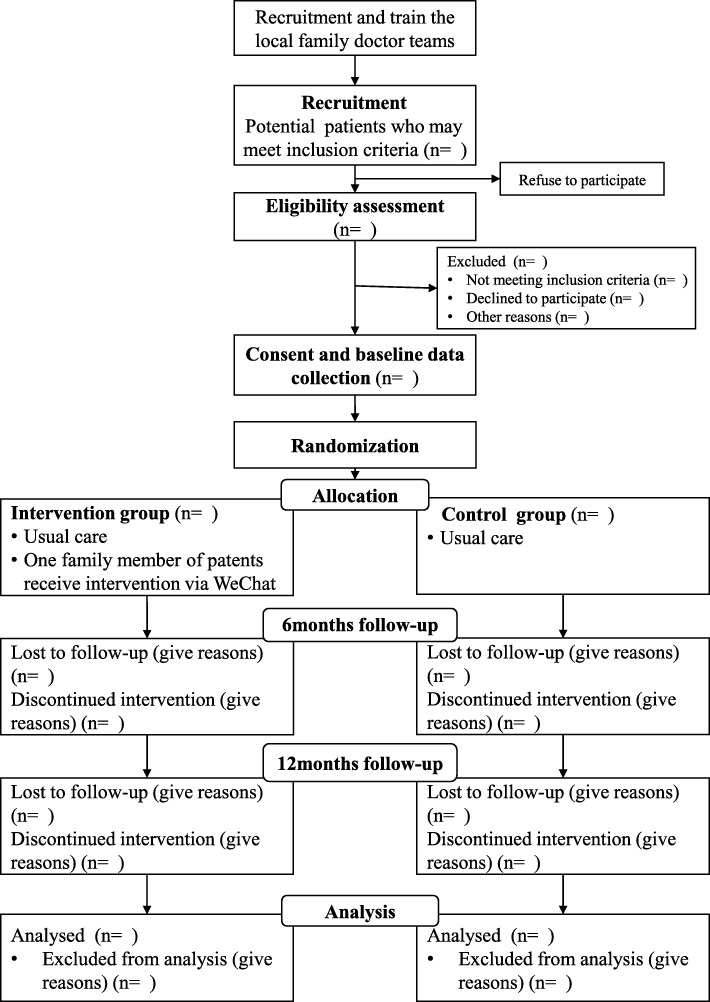
CONSORT flow diagram of the study design

A parallel, two-group, randomized controlled trial with equal allocation to the experimental group will be carried out over a year (Fig. 1). The participants will be randomly allocated to intervention group or control group. The participants in intervention group will receive the family-based intervention besides the usual care, while the participants in control group only receive usual care [8]. This study will be implemented and reported on the basis of Consolidated Standards of Reporting Trials (CONSORT) 2010 statement [30] and the Standard Protocol Items: Recommendations for Interventional Trials (SPIRIT) guidelines [31] (see Additional file 1).

The trial will be carried out in the central urban area of Jiading district in Shanghai, China. Jiading is located in the northwest of Shanghai. Per capita GDP of Shanghai in 2017 was 124,536 yuan, ranking it second among the 31 provinces in mainland China [32], while that of the whole mainland China was 59,660 yuan. The per capita GDP of Jiading in 2017 was 104,423 yuan, ranking it sixth among the 16 districts in Shanghai [33]. Nearly 150,000 residents live in this central urban area. Of them, 3873 are registered patients with type 2 diabetes according to the health information system of the basic public health services of the two health community centers in this area. Family doctor teams, usually consisting of one primary health doctor and 2–3 nurses, are responsible for providing the usual care stipulated by the national basic public health policy as their routine work. Diabetes self-management education (DSME), As an important part of usual care, is recommend to be held quarterly in the local community, consisting of basic diabetes information, how to practice self-monitoring of blood sugar, proper diet, how to inject insulin and other medications, physical activity, and foot care.

The blood glucose control compliance rate of registered diabetic patients from the two community health centers was 37.7% based on the available HbA1c data of 1650 patients in the health information system. This was close to the average rate in Shanghai city [34].

### Ethics and trial registration

The trial was ethically approved by Medical Research Ethics Committee at School of Public Health Fudan University (IRB#2018-01-0663), and the Trial Registration number is ChiCTR1900020736.

### Study participants

Both patients with poorly controlled type 2 diabetes and their designated family members will be participants in this study.

### Inclusion and exclusion criteria for patients

Inclusion criteria: 1) registered in the two community health service centers; 2) has had type 2 diabetes, diagnosed by a doctor at least 6 months before the study enrolment; 3) aged 18–79 years; 4) has HbA1c levels of ≥7.0%; 5) has no plans to leave home for the following 12 months; 6) has a family member who can use WeChat and lives with the patient or visits at least once a week.

Exclusion criteria: 1) has other serious illnesses or illnesses not suitable for this study; 2) women who are pregnant or preparing for pregnancy; 3) unable to complete 12-month follow-up; 4) unwilling or unable to provide informed consent; and 5) is currently participating in other intervention studies.

### Inclusion and exclusion criteria for family members

In this study we defined family members as relatives who contact with patients regularly. The family members will be nominated by patients. Patients need to choose one of his/her family members as a supporter to receive the corresponding intervention. The family member should be more than 18 years old and have no history of diabetes.

### Recruitment

The two community health centers consist of 12 community health service stations, with a family doctor team in each station. Twelve family doctor teams, responsible for the health management of people with type 2 diabetes in their routine work, will be recruited from the community health service stations. In this study, their tasks will include: mobilizing the participation of patients and their families and collecting baseline and follow-up data. After program training by researchers, they will commence the recruitment of potential participants.

Potential participants are those who have electronic medical records in the two community health centers. The family doctor team will call the patients with HbA1c data showing a value of ≥7.0%, explaining the objectives of the study and judging whether the patents are eligible to participate. In addition, due to the lack of HbA1c data for some patients in the database, the family doctor team will seek out potential eligible participants based on the data for fasting blood glucose during a patient’s last four follow-up visits that indicate poor glycemic control. The family doctor team will call the potentially eligible participants and explain the nature of the study. As part of eligibility assessment, all potential participants who are willing to participate will be asked to undergo HbA1c testing to make sure their HbA1c levels make them eligible.

Staff from the family doctor team will make an appointment for the eligible patients at the community health service station. At this appointment, patients and their family members can further consult project-related information. Once the patients and family members decided to participate, both will be asked to sign the consent form. Then, they will be asked to complete the baseline survey. The staff will keep a record the reasons for the eligible patients who reject to participate. In order to investigate sub-group differences, demographic variables, such as age and gender, will be compared between the participants and non-participants.

### Randomization

After collecting all baseline data, we will do the randomization at the individual level. Participants will be allocated to intervention or the control group in a 1:1 ratio. The randomization were performed by Stata 14.0 after all participants has been stratified by gender, duration of illness, and age. It is not possible to blind study researchers, the operator of the WeChat public account, or the participants due to the nature of the intervention. The community health service centers’ staff who are mainly responsible for collecting data, however, are blinded during the data collection.

**Fig. 2:**
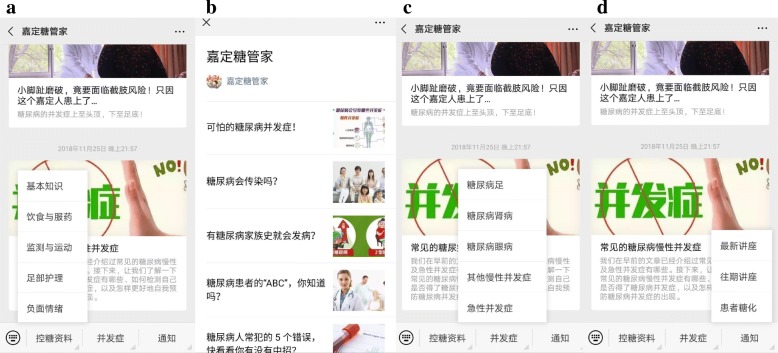
A sample interface of the Jiading Sugar Steward WeChat public account. Note: (**a**) The first module: information and skill; (**b**) Example content of the first module; (**c**) The second module: complications of diabetes; (**d**) The third module: lecture notice and a report about HbA1c value

All family members (not patients) from the intervention group will be invited to subscribe to the WeChat public account *Jiading Sugar Steward* on their smartphone (Fig. 2, the interface of this WeChat public account). This group will receive the usual care stipulated by the national basic public health policy. The controls will not be able to get access to the information of the WeChat public account.

### Intervention design

#### Conceptual model

**Fig. 3:**
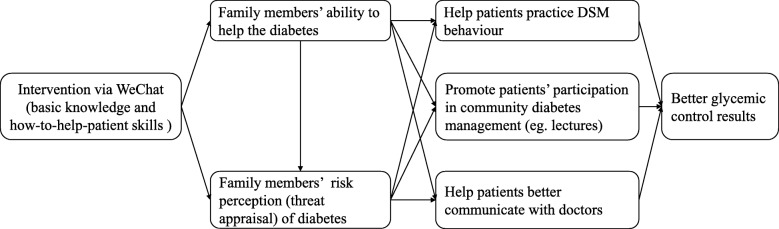
How family members may influence outcomes in diabetes

A conceptual model for how family members’ knowledge about diabetes may influence patients’ health outcomes is presented in Fig. 3. The intervention was developed mainly on the basis of the Health Belief Model (HBM) [35], which is widely used for changing health-related behavior. The theory indicates that motivation toward preventive behaviors results from threat appraisal and the desire to avoid a potentially negative outcome [36].

According to this model, education via WeChat will improve family members’ basic knowledge and skill. Therefore, they will have better ability to help the patients. In addition, they will have strong risk perception that patients with uncontrolled diabetes will suffer diabetes-related complications. As a result, both improved ability and risk perception will encourage and enable family members to take appropriate action in helping patients coping with diabetes. With the support of family members, patients may practice DSM with more confidence.

#### Information content

About 105 articles have been developed for the intervention based on *Guidelines for the Prevention and Treatment of Type 2 Diabetes in China-2017* [37]. A panel of five healthcare professionals of diabetes and/or patient health education will agree on the contents.

The article for this intervention will be divided into three main modules. The first module (Fig. 2a) consists of seven elements: 1) basic information about diabetes; 2) self-monitoring of blood sugar; 3) eating healthily; 4) insulin injections and medications; 5) reasonable exercise; 6) foot care; and 7) dealing with negative emotions. Information in this module covers basic knowledge about and the benefit of behavior change and how to practice it well. Common misunderstandings (Fig. 2b), such as that patients can stop medication when the blood sugar returns to normal, will be emphasized. Family members will be encouraged to help patients practice self-management behaviors and deal with negative emotions.

The second module (Fig. 2c) focuses on the possible complications of diabetes and dealing with emergencies like hypoglycemic coma and diabetic ketoacidosis. This information will give participants more details about the severity of diabetes and change the risk perceptions of family members.

The third module (Fig. 2d) is a news module consisting of two elements: a lecture notice and a report card containing the actual A1C value. The health education lecture notice will be released again in this module. We hope that the family member will encourage and remind the patients to attend all of the health education lectures. The report card will be presented with the patients’ current hemoglobin A1C and the ideal value. This report card will used as a form of feedback for family members to stimulate long-term social support.

#### Information delivery

To improve the accessibility of the intervention, the frequency and time of contacts must be considered. Too much information may bother participants. Thus, we decided to provide interventions twice a week. Although some studies have shown that WeChat users are more likely to receive push information from 8 a.m. to 11 a.m. and from 6 p.m. to 8 p.m. [38], there is no conclusion in terms of when is the most appropriate. Considering the working time of most people and the results of earlier studies [39], we will release the content at 5.00 p.m. to prompt the subscriber to accept the intervention. Due to the function of the WeChat application, the researcher can set the time to send the articles automatically. All information of this intervention can be conveyed by various methods such as text, images, videos, and audios.

#### Questions and quizzes

The participants will be encouraged to send their diabetes-related queries in the comments section after they have read the corresponding articles. The research staff, who are responsible for opening the public account, will collect queries every two days and send them to experts. Six experts (three from the Fudan University Zhongshan Hospital, two from the Jiading District Center for Disease Prevention and Control, one from Jiading Mental Health Center), as a consulting group, will address questions from participants.

In some important articles, quizzes will be included to improve the participants to actively read the articles and to assess how the participants master the information. The quizzes usually include 1–2 true-false or multiple choice items and is a closed test. To stimulate participants’ involvement, we will randomly select half of the quizzes and provide their takers with a monetary bonus. Participants who complete quizzes will get the bonus, usually 2–10 RMB, regardless of the correctness of the answer.

### Sample size

Sample size will be calculated based on the primary outcome, difference in mean HbA1c levels, assuming 80% power and a two-sided significance level of 0.05. The supposed effect size of HbA1c is 0.5%. We use the same standard deviation of HbA1C (1.2%) for calculations of the sample size, according to the data of 879 registered patients with HbA1C values more than 7.0% in the health information system in the two community health centers in central urban Jiading. Therefore, 92 participants are required in each group. Considering the possible lost follow-up rate of 20%, we will require 111 patients in each group for a total enrollment of 222 patient-family member dyads.

### Pilot study

A one-month pilot study was conducted to assess the feasibility and acceptance of the main study, gathering information which might be helpful for to improve the intervention design. Participants for the pilot study were recruited from the 5 community health centers in Jiading district. Using convenience sampling, 50 eligible patients (27 women and 23 men, mean age 62 years) were recruited in October 2018. Twenty-four of them (15 women and 9 men) and their family members agreed to participate in the pilot study. Patients were randomly and equally divided into intervention and control groups, with 12 in each group. Patients’ family members in the intervention group were invited to subscribe to the WeChat public account *Jiading Sugar Steward* for one month. Limited by time, we were unable to release all of our information via the WeChat account in the pilot study, but we chose to release 10 articles regarding different themes.

After a month, the data collected from the WeChat official accounts platform showed that the average number of these articles that were actually read was 3.83. Family members in the intervention group were called to complete a questionnaire and make suggestions. The most appreciated feature within WeChat was convenience: family members considered it a convenient way to receive information.

We also conducted a semi-structured interview with 6 family members. Respondents (*n* = 6) reported that they regarded this WeChat public account as a tool to learn diabetes-related knowledge.

*Though my mother has been diagnosed with diabetes for many years, I know little about this disease … and I am often confused with it. The articles from the WeChat public account I read are really helpful … I hope you can provide more information.* (A daughter)

*Mother often told me not to worry about her … and I have little knowledge about her diabetes. Sometimes I feel frustrated because I don’t know how to help her. Information on the internet is too messy … I have little time to attend community health education lectures … The WeChat public account provides more convenience.* (A son)

Some respondents (*n* = 2) also reported that they wanted more detailed information about healthy eating.

*Compared with other sorts of information, I want to have more understanding about how to eat. I think my father does well in other aspects, but not well in this.* (A daughter)

*Eating is closely related to daily life; the article you put on WeChat is too brief. I only have a vague conception of what food diabetics should not eat.* (A wife)

However, there was a problem with the acquisition of information. Some respondents (*n* = 3) reported that they often neglected the articles.

*And you know, I subscribe to many WeChat public accounts … .so I was unable to read every article from this public account.* (A daughter)

Based on the feedback from the pilot study, the intervention was revised. We added a WeChat personal account, named *Jiading Sugar Assistant*, as a supplement to deliver information to the participants individually. We also adjusted the content of the information, added more detailed information, and condensed knowledge.

### Outcome measures

The aim of this study is to improve clinical outcomes of patients with poorly controlled type 2 diabetes. HbA1c has been clinically used as a gold standard for assessing long-term glycemic control, reflecting over approximately 3-months blood glucose concentrations [40, 41]. Therefore, the primary outcome is the change in HbA1c of patients between at baseline and at the sixth month, and that between at baseline and the twelfth month.

Secondary outcome measures include changes in weight, height, waist circumference, hip circumference, blood pressure, BMI, blood glucose (fasting plasma glucose and 2 h post-load glucose), diabetes self-care activities, medication adherence, risk perception of diabetes, and family support. In addition, changes of family members’ diabetes-related knowledge and support to the patient are also assessed.

### Data collection

Data will be collected at baseline, 6 months, and 12 months for both patients and family members from the intervention group and the control group. For participants’ convenience, follow-up sessions will be scheduled based on the participants’ regular appointments, once every three months at the community health service stations.

Data collection will consist of two main parts: a questionnaire and biophysical data. Table 1 shows the measurement tools for patients’ data collection, the method and time points of data collection. The questionnaire data includes four main parts. The first part covers social demographics (e.g., sex, date of birth, education level, job, health insurance, marital status, annual income, family population composition, etc.). The second part concerns patients’ health status (e.g., time since diagnosis, therapeutic regimen, complications and co-morbidities, and hospital visit status due to diabetes). The third part is diabetes self-management behavior including activities of self-management [42] and adherence to medication [43]. The fourth part is psychosocial status, including risk perception [44], perceived family support [45], diabetes-related knowledge, and perceived benefits and barriers to diabetes self-care.

**Table 1:** Patients’ variables and measurement tools used at each data collection time point

Variable	Measurement tools/questions	Baseline	6 mth	12 mth
Demographic measures	Sex, date of birth, ethnicity, education level, job, health insurance, marital status, annual income, family population composition	**√**		
Health status	Duration, therapeutic regimen, complications and co-morbidities, hospital visits due to diabetes	**√**		**√**
Behavior	Adherence to medication (Morisky Scale), activities of self-management (SDSCA)	**√**	**√**	**√**
Psychosocial variables	Risk Perception Survey–Diabetes Mellitus (RPS-DM), perceived family support (DFBC), diabetes-related knowledge, source of diabetes-related knowledge, perceived benefits and barriers to diabetes self-care	**√**	**√**	**√**
Biomedical measures	HbA1c, weight, height, waist circumference, hip circumference, blood pressure, BMI, blood glucose (fasting plasma glucose and 2 h post-load glucose)	**√**	**√**	**√**

The biophysical data, except for HbA1c, can be obtained from the medical record, including: weight, height, waist circumference, hip circumference, blood pressure, BMI, and blood glucose (fasting plasma glucose and 2 h post-load glucose). All these data will be collected as part of the family doctor team’s work. For the data on HbA1c, blood specimens will be collected from patients needing such testing by specifically trained staff according to standard procedures, then sent to the approved laboratory for analysis.

The questionnaire for collecting data from family members includes three main parts and will be administered by trained investigators. Table 2 shows that the first part covers social demographics (e.g., sex, age, ethnicity, religion, education level, job, health insurance, marital status, annual income, relationship with patients, etc.). The second part is health status (e.g., family history, chronic disease). The third part is family support and knowledge of diabetes.

**Table 2:** Family members’ variables and measurement tools used at each data collection time point

Variable	Measurement tools/questions	Baseline	6 mth	12 mth
Demographic measures	Sex, age, ethnicity, education level, job, health insurance, marital status, annual income, relationship with patients	**√**		
Health status	Family history, chronic disease	**√**		
Family support and knowledge of diabetes	Family support (DFBC), diabetes-related knowledge, source of diabetes-related knowledge	**√**	**√**	**√**

The patients and their family members will be given linked ID numbers. The first three digits of the patient and his/her family member in the dyad will be the same, but the patients’ ID will end with the number “1” and family members’ ID will end with the number “2.”

### Data analysis

Descriptive statistics will be applied to describe the study population at baseline. We will analysis the categorical variables such as sex, job using frequency distribution description and centralized trend description. We will analysis the continuous variable using means±standard deviations. We will compare the difference of characteristics of participants (patients and family members) by t test or chi-square 2 test or non-parametric equivalent test. The *p* value 0.05 will be considered as the level of significance.

Effectiveness of the intervention will be measured by the differences primary outcome and secondary outcomes measures between study groups. The data about primary outcome and secondary outcomes are longitudinal data, thus we will use generalized linear mixed models. Potential confounders and effect modifiers (e.g., gender, age, and income) will be investigated.

Process data will consist of three parts, WeChat public account data, participation in diabetes self-management education, and an interview. The data from the WeChat public account mainly include: participants in the intervention who canceled their subscription, reading quantities of various sorts of articles (different information content and delivery methods), comments and questions. Patients’ participation in education will be recorded, and we will compare the difference between the intervention group and the control group.

Quantitative data will be analyzed with STATA using descriptive statistics. Semi-structured interviews of patients, family members, and staff of the family doctor teams will be analyzed with NVivo.

## Discussion

This trial is designed to improve clinical outcomes of people with poorly controlled type 2 diabetes. Based on our knowledge, this is the first study to assess the effectiveness of delivering a family-based intervention via WeChat for people with diabetes in the community.

This study has two major strengths: family involvement and delivery via a convenient and accessible tool—WeChat. There is a close relationship between patients with diabetes and their family members, with patients relying heavily on their families for decision-making [13] even though many family members do not know how to help the diabetic [21]. Therefore, this intervention is expected to generate ongoing support from family members toward patients. The free application, WeChat, provides us with the opportunity to solve the problem of time and distance requirements. It will also empower the participants by providing them with total control over the intervention materials.

The biggest challenge we have encountered since beginning this study is a low participation rate, though the pilot study showed that 48% of eligible patients consented to participate in the trial, which is much higher than the rate in previous studies [21, 22]. There are various reasons for this, the most common being that patients are not willing to trouble their family members. To solve this problem, it is necessary that the family doctor teams explain the usefulness of the family members’ participation. If the intervention is proven to be effective, this study would provide evidence that educating family members via WeChat is an effective and feasible way to improve glycemic control in patients with patients with type 2 diabetes. Furthermore, this intervention can be popularized and implemented in areas with similar population economic characteristics by the implementation of basic public health service.

There are limitations in this study. We will be conducting this RCT in the central urban area of Jiading district, which means the participants (especially family members) may be of a higher socioeconomic background than the average Chinese patient. They may not be representative of the entire population, especially for people in rural China. Like all web-based interventions, this study also faces the problem of high dropout rates [46]. In order to maximize participation, we will be regularly reinforcing it by providing bonuses.

## Trial status

The pilot study was from October to November 2018, and, with minor adjustment, the intervention design was completed in December 2018. The recruitment of participants and baseline assessments started in January 2019 and are expected to be completed in February 2019. The formal intervention will be started in March 2019.

## Additional file

Additional file 1: SPIRIT 2013 Checklist: Recommended Items to Address in a Clinical Trial Protocol and Related Documents. (DOC 91KB)

## Abbreviations

BMI: body mass index
DFBC: diabetes family behavior checklist
DSM: diabetes self-management
DSME: diabetes self-management education
HbA1c: glycated haemoglobin
